# Does the Flipped Classroom improve exam performance in medical education? A systematic review

**DOI:** 10.15694/mep.2017.000100

**Published:** 2017-06-13

**Authors:** Yasmin Hughes, Nicholas Lyons

**Affiliations:** 1 University of Queensland; 2 Prince Charles Hospital

**Keywords:** Flipped classroom, Medical education, Undergraduate, Postgraduate

## Abstract

This article was migrated. The article was marked as recommended.

***Background:*** The ‘flipped classroom’ (FC) is a blended learning model in which educational material is delivered on-line prior to class, students then apply this knowledge through discussion and problem solving activities in class. Its effectiveness in medical education is debated. The aim of this systematic review is to assess the outcomes of studies which have compared the exam performance of traditional didactic teaching (DT) to the FC in medical education.

***Methods:*** A broad based literature search was performed in accordance with PRISMA protocol. MESH terms were ‘flipped classroom’, ‘flipped teaching’, ‘blended learning’ and ‘medical education’. The outcomes of interest were test score results following FC compared to DT methods.

***Results:*** Eleven studies with a total of 2052 study participants were included in the review. Four studies demonstrated a significant improvement in test scores using FC compared to DT; four showed no significant difference and three demonstrated mixed results.

***Discussion and Conclusions:*** The results of this review are equivocal. Study heterogeneity in design, participants and subject covered may account for some of this disparity. Two studies provide evidence that the FC results in improved performance on higher cognitive tasks however further robust, in depth studies are required to demonstrate this conclusively.

## Introduction

Medical education is faced with training medical students and doctors to become competent life-long learners at a time when medical knowledge is increasing at an exponential rate. Just as medical knowledge is advancing, so too is technology. To address the challenge faced by medical education, there has been growing interest in innovative approaches using on-line learning platforms. Whilst distance learning has enjoyed success in a number of formats ( Kay, Reimann, & Diebold, 2013) it is unlikely that online, distance learning courses will be able to entirely replace face to face medical teaching for several reasons. Firstly, practicing medicine relies on excellent communication skills; skills that are unlikely to be developed with an on-line resource. Secondly, there is proposed benefit in learning collaboratively. ‘Communities of practice’ defined by organisational theorist Etienne Wenger as “groups of people who share a concern, a set of problems, or a passion about a topic, and who deepen their knowledge and expertise in this area by interacting on an ongoing basis,” has merits in medical education ( Wenger, 1998). Thirdly, development of complex reasoning skills is at the core of medical education ( Connor et al., 2016). Collaborative face to face ‘active’ learning offers the opportunity for students to engage in higher order cognitive tasks such as application, evaluation and synthesis of knowledge ( Connor et al., 2016; Moffett & Mill, 2014), skills which are essential in a practicing clinician.

“Blended learning”, the combination of teaching delivered by on-line digital media with face to face learning potentially provides a solution to balancing the use of technology to assist in delivering large volumes of medical information with hands on clinical coaching. The ‘flipped classroom’ (FC) is a student centred, blended pedagogical model in which the homework and classroom components of a teaching session are reversed. In this type of blended learning, students work independently, prior to class, to learn basic facts and concepts through on-line educational content such as on-line videos, quizzes or podcasts. As the students are prepared prior to entering class, classroom time is devoted to the application of this knowledge through discussion, problem solving and deeper probing of concepts. This approach has been widely accepted by a number of disciplines ( Adams, Garcia, & Traustadóttir, 2016; McLaughlin et al., 2013; Moffett & Mill, 2014).

Conceptually, the FC is well suited to medical students who, in large, are highly motivated, independent learners. The opportunities for discussion of concepts and interactive application of knowledge to clinical problems, whilst promoting higher order cognitive thinking, simulates clinical working environments. Since its development to deliver a core biochemistry course at Stanford School of Medicine in 2012 ( Prober & Heath, 2012), a number of prestigious institutions have adopted this approach to delivering medical education, including Harvard School of Medicine ( Fu & Joung, 2015). This type of learning is also suited to postgraduate training doctors where teaching time needs to fit around demanding clinical commitments. Yale School of Medicine is currently using this concept to deliver anaesthesia residency training ( Yale School of Medicine, 2017).

Despite its implementation, within both undergraduate and postgraduate medical education settings, little is known about the effectiveness of the FC in terms of objective quantitative measures and how these outcomes compare to traditional didactic teaching (DT) approaches. A body of research has investigated the qualitative effects of the FC and a recent systematic review addressed medical students perceptions of the FC ( Ramnanan & Pound, 2017). Whilst this is of interest, it would be beneficial to evaluate learning (level two) by students rather than just reaction (level one) as per Donald Kirkpatrick’s four levels of evaluation model ( Kirkpatrick, 2007). The recent review addressed undergraduate medical education, however, failed to appreciate the importance of evaluating the effect of the FC in postgraduate doctor training. At the time of writing, no systematic reviews exploring the FC and medical education have specifically considered whether the FC results in improved examination performance compared to DT methods. Moreover, no reviews to date have considered the effects of the FC in both undergraduate and postgraduate medical education settings. We aim to rectify this.

The main objective of this systematic review is to investigate and critically appraise the published data on quantitative outcomes of the use of the FC compared to DT approaches in both undergraduate and postgraduate medical education.

## Materials and methods

A literature search was performed in accordance with the Preferred Reporting Items for Systematic Reviews and Meta-analysis (PRISMA) protocol. MeSH search terms used were ‘flipped classroom’, ‘flipped learning’, ‘blended learning’ and ‘medical education’; terms were combined using ‘and/’or’ as appropriate. These terms were then applied to Medline (since 1952), EMBASE (since 1980), the Cochrane library (since 1995), CINAHL (since 1982) and Google Scholar and the search conducted independently by the authors. MeSH terms were exploded to identify additional studies.

### Inclusion criteria

Included studies assessed the effectiveness of the FC when used in medical education (pre or post graduate) and compared reported objective test score results for FC to scores achieved with DT. FC was defined as an educational technique that consisted of two parts: computer-based individual instruction that occurred both prior to class and outside of the classroom, and interactive group learning activities which occurred during classroom time. All studies including this design were included irrespective of their use of the term FC. Studies were carefully evaluated for duplication or overlapping data.

### Exclusion criteria

Studies were excluded that did not use a FC teaching modality as determined by the authors, did not specifically study medical student or doctor education, or failed to provide quantitative objective outcomes (assessment results). Medical education was determined as modern western medicine only. Chinese, Ayurveda and traditional medical practises were excluded on the basis of large heterogeneity in underlying philosophies and factual knowledge likely to result in a confounded comparison. Letters, reports, conference abstracts or abstract only reports were excluded.

### Outcome measures

Outcomes of interest were assessment score results post a FC intervention compared to post DT.

### Study selection

The authors independently performed the search strategy initially undertaking a title screen followed by abstract review and full text review of appropriate studies. Publications satisfying the exclusion criteria were discarded at each stage ( figure 1). Publications without abstracts moved straight to full text review. Discrepancies between author searches were resolved by consensus following discussion.

### Data extraction

Data extraction was undertaken independently by the authors using a standardised proforma for the objective scores only. The following demographic and medical education parameters were extracted from each study; study characteristics (first author, year of publication, study design, Institution), student characteristics (number and demographics), outcome measures (assessment method, quantitative results, qualitative results).

### Quality assessment

Quality assessment was undertaken using a Modified Index for Non-Randomised Studies (MINORS) ( Slim et al., 2003) modified for a non-clinical research question (see appendix). Specifically, point two (Inclusion of consecutive patients) was not relevant to any of the studies included in the final review and was thus omitted. The remaining eleven items were used as per original description. Prospective design criteria was applied to the comparison group only.

### Publication bias

No statistical analysis assessing the risk of publication bias was undertaken as per guidance in the “Cochrane handbook of systematic reviews of interventions” ( Higgins & Green, 2011). Studies of eleven or fewer articles do not require analysis for publication bias.

## Results

The initial search returned 345 articles after removal of duplicates. 225 of which were excluded at title screen. 120 articles were taken forward for abstract review, 94 articles were excluded (see figure 1). The remaining 26 articles went forward to full text review, of which 15 were excluded (nine failed to compare FC and DT, five provided inadequate objective test results, one using Chinese medicine) leaving eleven studies ( Belfi, Bartolotta, Giambrone, Davi, & Min, 2017; Bonnes et al., 2017; Boysen-osborn et al., 2016; Connor et al., 2016; Evans et al., 2016; Gillispie, 2016; Heitz, Prusakowski, Willis, & Franck, 2015; Liebert et al., 2017; Morton & Colbert-getz, 2016; Rui et al., 2017) which were included in quantitative analysis.

A total of 2052 students were included in the analysis, 1098 of which underwent the FC condition and 954 in the DT group. Of the DT group, six (14-16,18,19,22) used a retrospective cohort as a comparator group with the remaining five (12,13,16,17,20) studies using a contemporary DT control group.

### Allocation of groups

Of the five Contemporary case control studies, two studies randomised students to either the intervention group (FC) or control group (DT), two studies did not state how assignment was carried out and one study assigned students according to their post-graduate year of training (PGY). In each of the six Retrospective case comparison studies, a contemporary intervention (FC) group was compared to a retrospective control (DT) group.

### Contemporary case-control design

Of the five studies using a contemporary DT comparator group, three studies ( Belfi et al., 2017; Bonnes et al., 2017; Gillispie, 2016) used a simple case control design with students undergoing either FC or DT teaching. The remaining two ( Connor et al., 2016; Heitz et al., 2015) studies used a crossover design where students experienced both DT and FC conditions covering different teaching material for the two conditions. The study participants were either undergraduate or postgraduate medical students or postgraduate doctors. Medical students were enrolled in the first to fourth years and participating in either an Emergency medicine clerkship, Radiology clerkship or Pre-clinical ECG interpretation module. Postgraduate doctors were in either their first, second or third year of internal medicine training (see table 1).

### Retrospective case-control design

Six studies ( Boysen-osborn et al., 2016; Evans et al., 2016; Gillispie, 2016; Liebert et al., 2017; Morton & Colbert-getz, 2016; M. R. Sajid et al., 2016) used a retrospective comparator group, typically comparing FC test scores to those of previous years test scores following DT. Subject areas included in these six studies were; haematology undergraduate teaching, Obstetrics and gynaecology clerkship, Advanced cardiac life support teaching of medical students, Anatomy teaching and a Clinical epidemiology and biostatistics module delivered to first year medical students and a core surgery clerkship of third year medical students. Three of the retrospective case comparison studies ( Evans et al., 2016; Morton & Colbert-getz, 2016; M. R. Sajid et al., 2016) used multiple choice question (MCQ) exam alone with two studies using MCQ combined with another assessment method such as an Objective structured clinical examination (OSCE), questions regarding clinical cases or ECG rhythm strip interpretation ( Boysen-osborn et al., 2016; Gillispie, 2016). One study used pre and post intervention National Board of Medical Examiners (NBME) test scores as the quantitative assessment ( Liebert et al., 2017).

### Test scores

Contemporary case-control: There was variation in both the methodology and results of assessment within the contemporary case-control group. All studies assessed the teaching methods by examination scores; three studies used MCQ scores ( Belfi et al., 2017; Connor et al., 2016; Heitz et al., 2015), one study used a pre and post course QI knowledge assessment tool (QIKAT) ( Bonnes et al., 2017) and one study used an ECG interpretation examination ( Rui et al., 2017). Four of the five studies demonstrated a clear, statistically significant, improvement in assessment scores for the FC groups over the DT ( Belfi et al., 2017; Bonnes et al., 2017; Connor et al., 2016; Rui et al., 2017) with one study demonstrating no improvement for FC over DT ( Heitz et al., 2015). Two of the studies used a cross-over design with students experiencing both DT and FC conditions in an emergency medicine clerkship and radiology clerkship ( Connor et al., 2016; Heitz et al., 2015). These studies shared similar designs with two groups of students experiencing four modules, two of which were FC and two DT. Both were assessed with MCQs of 40 and 30 questions respectively. The results demonstrate no significant difference in performance for the emergency medicine clerkship whilst a significant improvement of 10.5% (p=0.013) was noted for the radiology clerkship study.

Retrospective case control: There was more variation in the results of the retrospective case-control group than noted in the contemporary case-control group. Of the six studies, three demonstrated no significant difference between FC and DT interventions ( Evans et al., 2016; Liebert et al., 2017; M. R. Sajid et al., 2016). Three studies demonstrated mixed outcomes. One study showed significant improvement in MCQ performance but no benefit to rhythm strip interpretation with both groups scoring 100% ( Boysen-osborn et al., 2016). Another showed improvement on OSCE performance with the FC but only 1/2 groups benefitting from FC on MCQ performance ( Gillispie, 2016). One study implemented a FC approach to teaching Anatomy and assessed students with an MCQ exam. The MCQs were characterised according to Blooms taxonomy to assess students at different cognitive levels including knowledge, application and analysis of anatomy items. They found that students in the DT group performed better in knowledge and application anatomy items whereas students in the FC group had higher scores on analysis questions.

### Statistical Analysis

Given the heterogeneity of the studies, the pooling of data for meta-analysis was deemed as inappropriate.

### Quality assessment

Studies were evaluated using a modified MINORs risk of bias tool. Of a maximum score of twelve points, studies ranged from five to ten ( appendix 1). One study ( Bonnes et al., 2017) scored ten, four scored eight ( Belfi et al., 2017; Boysen-osborn et al., 2016; Heitz et al., 2015; Rui et al., 2017), two scored seven ( Connor et al., 2016; Gillispie, 2016), three scored six ( Liebert et al., 2017; Morton & Colbert-getz, 2016; M. S. Sajid, Bokhari, Mallick, Cheek, & Baig, 2009) and one scored five ( Evans et al., 2016). Whilst overall performance on study end point, unbiased assessment and clear aims were good, no studies undertook prospective power calculations and with the exception of two studies ( Bonnes et al., 2017; Boysen-osborn et al., 2016), loss to follow up or failure to report total number of students completing examinations was high. A prospective design was undertaken by six of the eleven studies ( Belfi et al., 2017; Bonnes et al., 2017; Connor et al., 2016; Gillispie, 2016; Heitz et al., 2015; Rui et al., 2017).

## Discussion

Since the widely cited article describing the use of the FC to teach a core biochemistry course at Stanford Medical School in 2012 ( Prober & Heath, 2012), the FC has been introduced to deliver a wide range of medical curricula ( Fu & Joung, 2015; Yale School of Medicine, 2017). Previous reviews have presented qualitative outcomes demonstrating that the FC is preferred over DT in undergraduate medical education ( Ramnanan & Pound, 2017). This is the first systematic review to assess the effectiveness of the FC compared to traditional DT with objective test scores in both undergraduate and postgraduate medical education. Eleven studies were included in this review of which only four demonstrated a significant improvement for the FC over DT and a further three demonstrating what we have called a mixed outcome where more than one assessment was undertaken, some of which demonstrated benefit of FC and others not.

This variation is likely the result of several factors, the most notable of which are the variation in study design, student type and stage of learning, course material covered, study methodology and the type of assessment used ( table 1). In spite of this variation there are still some points of interest. Three studies ( Boysen-osborn et al., 2016; Gillispie, 2016; Morton & Colbert-getz, 2016) used more than one type of assessment in their design and reported mixed results suggesting that FC may be better for some types of learning but not others. One of the proposed benefits of the FC is that it allows students to engage in higher order cognitive tasks such as application, analysis and synthesis of knowledge ( Lampinen & Arnal, 2009). In their study, Morton et al, used assessment items in the form of MCQs which tested different cognitive levels of Bloom’s taxonomy including knowledge, application and analysis of Anatomy content. This study found that students taught Anatomy by the FC outperformed students taught by a lecture based method on MCQs that tested analysis however, a DT approach resulted in higher MCQ scores based on knowledge and application ( Morton & Colbert-getz, 2016). This supports the notion that, in terms of Bloom’s revised taxonomy, traditional lectures promote lower level cognitive work (gain and comprehension of factual knowledge), whereas the FC offers the opportunity for students to engage in higher order cognitive activities such as analysis, evaluation and synthesis of knowledge ( Ramnanan & Pound, 2017). This may explain the mixed results from other studies.

Gillipsie et al ( Gillispie, 2016) found that the FC students outperformed the DT cohort in OSCEs however, there was a statistically significant increase in MCQ scores for some topics delivered by a DT approach. The OSCE is a well-established mode of assessment specifically designed to provide a valid and reliable measure of students clinical competence ( Terry, Hing, Orr, & Milne, 2017). It requires higher order clinical reasoning skills that go beyond basic factual recall of knowledge. One could argue that, whilst the DT method may have resulted in improvement in factual recall in MCQ topics, FC resulted in improvement in higher order problem solving and clinical reasoning skills which are assessed by an OSCE. These are interesting observations that would benefit from further investigation.

The concept of the FC relies on students doing their homework. As this preparation forms the basis for basic knowledge acquisition, in its absence, the class time opportunities for application of knowledge and deeper probing of concepts become redundant. Few studies documented compliance with use of the online material. Heitz et al illustrated no significant difference between FC and DT groups during an Emergency medicine clerkship, however, almost one third (31.1%) of students stated that they were unable to adhere to the study protocol. However, when including data from only students who had followed the protocol, the authors found no statistical difference between FC and DT scores (p=0.8071). Therefore, the level of student concordance alone is unlikely to account for these results.

There are a number of limitations to this systematic review. As discussed above, the number of studies is small, only eleven in total, with large heterogeneity. Clearly there is a need for larger well designed studies to clearly demonstrate whether there should be a place for the FC in medical education and if so, where.

## Conclusion

There has been extensive research demonstrating the FC is preferred by students over DT, we would argue that the evidence presented in this systematic review only demonstrates superiority of FC over DT in higher cognitive tasks and does not clearly demonstrate any benefit from widespread use of FC in medical education. More robust studies are required to clearly demonstrate the role of FC in medical education.

## Take Home Messages

• The Flipped Classroom has been incorporated into a number of medical school programs and postgraduate medical training programs
• Studies of its effectiveness to date have largely been centred around qualitative student outcomes
• A review of quantitative outcomes shows mixed outcomes of its effectiveness when applied to medical education
• More robust studies are required to clearly demonstrate the role of FC in medical education.

## Notes On Contributors

**Yasmin Hughes MBiochem, MBChB** is a Lecturer in Clinical Science at the University of Queensland, Brisbane.

**Nicholas Lyons BSc (Hons), MBChB, MRCS** is a registrar in General surgery at Prince Charles Hospital, Brisbane.

## Figures and Tables

**Table 1.**
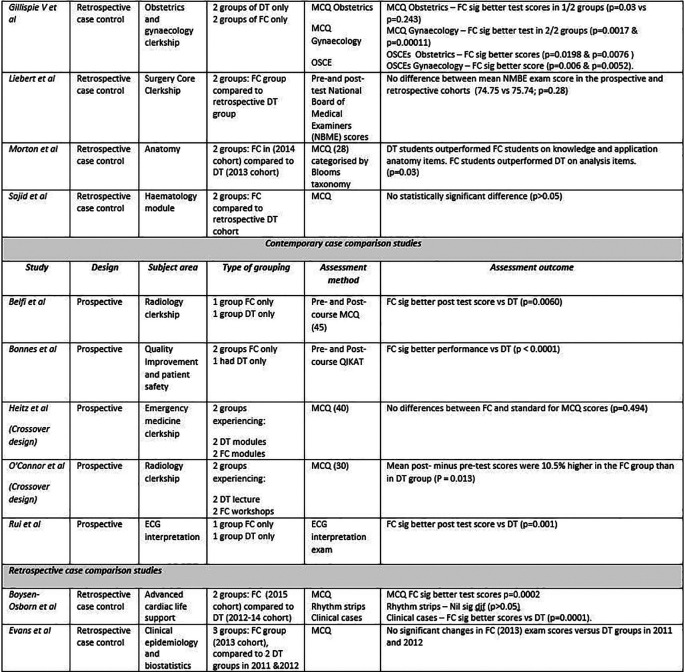
Summary of quantitative results

## Appendices

**
Figure 1 : PRISMA Flow chart**

### Appendix 1 Modified MINORS risk of bias assessment tool

**Table:**

Study	Clear aim	Prospective design	Appropriate end point	Unbiased assessment end point	Lost to follow up (<5%)	Prospective calculation of study size	Total score
*Belfi et al*	2	2	2	2	0	0	8
*Heitz et al*	2	2	2	2	0	0	8
*Boysen-Osborn et al*	2	0	2	2	2	0	8
*Evans et al*	1	0	2	2	0	0	5
*Gillispie et al*	1	2	2	2	0	0	7
*Liebert et al*	2	0	2	2	0	0	6
*Morton et al*	2	0	2	2	0	0	6
*O’Connor et al*	1	2	2	2	0	0	7
*Sajid et al*	2	0	2	2	0	0	6
*Bonnes et al*	2	2	2	2	2	0	10
*Rui et al*	2	2	2	2	0	0	8
