# Supplementary material for: Does the Flipped Classroom improve exam performance in medical education? A systematic review
Source: MedEdPublish (2016). 2017 Jun 13;6:100. [Version 1] doi: 10.15694/mep.2017.000100 (PMC10885262; doi:10.15694/mep.2017.000100)
Supplement: Peer review history

# Reviewer report: Reviewer response for version 1

**DOI:** 10.21956/mep.19262.r27987

**Published:** 2017-06-15

**Authors:** BALAJI ARUMUGAM

**Affiliations:** 1 TAGORE MEDICAL COLLEGE AND HOSPITAL

This review has been migrated. The reviewer awarded 3 stars out of 5 The author has made a great initiative in publishing a systematic review in an appropriate way on assessing the effectiveness of a newer and growing teaching learning method (FC). Although many institutes utilize or adapt different TL methods, generally the effectiveness or the outcome was not clearly expressed in individual studies. Or most of the time the investigator will find out that the newer TL method has always given a better result than the traditional methods. But adapting these newer methods in regular curriculum is the challenge. “The overall evidence does not clearly demonstrate any benefit from widespread use of FC in medical education”, which questions the utilization of newer TL methods.

Reviewer Expertise:

NA

No decision status is available

# Reviewer report: Reviewer response for version 1

**DOI:** 10.21956/mep.19262.r27989

**Published:** 2017-06-13

**Authors:** P Ravi Shankar

**Affiliations:** 1 American International Medical University

This review has been migrated. The reviewer awarded 4 stars out of 5 Thank you for the opportunity to review this interesting manuscript. Flipped classrooms (FC) are becoming increasingly common in education and are in consonance with modern concepts of education. Obtaining objective evidence of efficacy in educational interventions is more challenging due to a variety of reasons. In the Materials and Methods section, end of paragraph 1: Can the authors clarify the sentence ‘MeSH terms were exploded to identify additional studies.’ The authors have clearly defined the criteria used during their systematic review. There may be problems in using retrospective data to compare flipped classrooms and didactic teaching methods as the two groups of students may have different demographic and other characteristics. The authors have described the strengths and limitations of their study in detail. As medical educators we may have to discuss the importance of different levels (hierarchy) of knowledge, skills and attitudes in medical education. The authors have mentioned that FC is more effective in developing higher order cognitive skills. Accessing knowledge has become easier with the advent of the internet and other technologies and critical analysis and judgment skills are becoming increasingly important. This has influenced the use of different learning methods in medical education. The tables and the figures are well designed and supplement the information provided in the text. As mentioned by the authors future studies may be able to provide a more definitive answer with regard to the influence of the flipped classroom on exam performance.

Reviewer Expertise:

NA

No decision status is available

# Reviewer report: Reviewer response for version 1

**DOI:** 10.21956/mep.19262.r27988

**Published:** 2017-06-13

**Authors:** Trevor Gibbs

**Affiliations:** 1 AMEE

This review has been migrated. The reviewer awarded 2 stars out of 5 There is little doubt that we need to constantly evaluate the real effects of new teaching techniques upon the students; hence this paper which was based upon a literature review covered an important area.However, I would propose that this paper addresses several issues that we still are unsure about;The flipped classroom is and was designed as a teaching methodology that moved the students fro a passive learning environment to an active one. It highlighted and attempted to solve the problem of addressing key problems around didactic learning such as poor cognition from the students, a superficial learning approach whereby students were not given enough time to ask specific questions to improve their learning and an attempt to promote deeper learning by improving relevance and integration through application of new knowledge. I am not sure that the pre-learning only involved on line learning- there are many ways of delivering pre-learning without using on-line technology.Is it enough that we use only quantitative methods to evaluate this new learning when indeed the basis of the flipped classroom is about assisting the student in their new knowledge and trying to improve their internal motivationShould we really be basing our evaluation on level 2 Kirkpatrick when we are really hoping that the new teaching method improves longer term retention of knowledge and application of that new knowledgeI was also worried somewhat about the search methodology:- why exclude qualitative papers- why are we not searching for papers relating to higher order cognition- why only medical education when there are so many papers delivered from other health professionals- why exclude/ not include the grey literature, particularly since this isa relatively new subjectI think that this paper, whilst useful, certainly leaves the reader with some doubts

Reviewer Expertise:

NA

No decision status is available
